# Telehealth or in-person HIV care? Qualitative study findings on decision-making from people with HIV and HIV care providers in South Carolina during the COVID-19 pandemic

**DOI:** 10.1371/journal.pdig.0000812

**Published:** 2025-04-08

**Authors:** Valerie Yelverton, Salome-Joelle Gass, Daniel Amoatika, Christopher Cooke, Jan Ostermann, Nabil Natafgi, Nicole L. Hair, Bankole Olatosi, Otis L. Owens, Shan Qiao, Xiaoming Li, Caroline Derrick, Sharon Weissman, Helmut Albrecht

**Affiliations:** 1 Department of Health Services Policy and Management, University of South Carolina, Arnold School of Public Health, Columbia, South Carolina, United States of America; 2 Division of Infectious Diseases, Department of Medicine, Duke University School of Medicine, Durham, North Carolina, United States of America; 3 Department of Epidemiology and Biostatistics, University of South Carolina, Arnold School of Public Health, Columbia, South Carolina, United States of America; 4 Department of Medicine, University of South Carolina, School of Medicine, Columbia, South Carolina, United States of America; 5 South Carolina SmartState Center for Healthcare Quality (CHQ), University of South Carolina, Columbia, South Carolina, United States of America; 6 Duke Global Health Institute, Duke University, Durham, North Carolina, United States of America; 7 College of Social Work, University of South Carolina, Columbia, South Carolina, United States of America; 8 Department of Health Promotion, Education, and Behavior, University of South Carolina, Arnold School of Public Health, Columbia, South Carolina, United States of America; 9 Department of Internal Medicine, University of South Carolina, School of Medicine, Columbia, South Carolina, United States of America; 10 Prisma Health Midlands, Columbia, South Carolina, United States of America; Iran University of Medical Sciences, IRAN, ISLAMIC REPUBLIC OF

## Abstract

The COVID-19 pandemic disrupted HIV care services across the United States. Telehealth was rapidly implemented to ensure HIV care continuity. Despite the evidence of unequal telehealth uptake among some people with HIV (PWH), the decision-making processes to determine who received telehealth or in-person care are under-researched. This study assessed which decision criteria and processes determined which HIV care visit type was used by PWH and HIV care providers during the COVID-19 pandemic. Qualitative in-depth interviews with 18 PWH and 10 HIV care providers from South Carolina assessed PWHs’ and HIV care providers’ decision-making criteria and processes for telehealth HIV care during the COVID-19 pandemic. Interviews were analyzed using thematic analysis. Most PWH (11 out of 18) and all providers had used telehealth for HIV care. To guide visit type decisions, interviewees reported decision-making criteria across four domains: patient-related criteria, clinical criteria, provider preference, and HIV care continuity. Patient-related criteria included patient preference, convenience, fear of COVID-19 exposure and stigma, and transportation barriers. Clinical criteria included the need for a physical exam, a person’s care history and health status. While all identified decision criteria were important, we found a hierarchical structure: care continuity superseded other criteria. Some clinical criteria were reported as decision-relevant criteria by providers but not PWH. Most PWH reported that they were included or took the lead in the visit type decision process. Decision-making processes to determine PWHs’ HIV care visit types considered criteria across multiple domains. The superseding criteria was to sustain HIV care continuity. To guide future telehealth use, shared decision-making is needed to weigh patient-related, provider-related, and clinical decision criteria and maintain care continuity, and to comprehensively include all relevant decision criteria.

## Author summary

Telehealth was rapidly implemented in human immunodeficiency virus (HIV) care during the COVID-19 pandemic. Currently, the decision-making processes to determine who received telehealth or in-person HIV care are under-researched. Therefore, we assessed which decision criteria and processes determined which HIV care visit type was used in HIV care during the COVID-19 pandemic. We conducted 28 in-depth interviews with 18 people with HIV (PWH) and 10 HIV care providers from South Carolina asking about decision-making criteria and processes for telehealth HIV care during the COVID-19 pandemic. All interviews were analyzed using thematic analysis. We learned from our interviewees that they used decision-making criteria across four domains: patient-related criteria, clinical criteria, provider-related criteria, and HIV care continuity. Patient-related criteria included patient preference, convenience, fear of COVID-19 exposure and stigma, and transportation barriers. Clinical criteria included the need for a physical exam, a person’s care history and health status. Decision criteria were structured in a hierarchy, with HIV care continuity superseding all other criteria. We conclude that to guide future telehealth use, shared decision-making is needed to comprehensively include all relevant decision criteria and to systematically and intentionally include PWH in the decision process to support their care satisfaction.

## Introduction

During the COVID-19 pandemic, people with HIV (PWH) in South Carolina (SC), an *Ending the HIV Epidemic* (*EHE*) priority state with high rates of new HIV diagnoses and rural cases [1,2], faced high levels of HIV care interruptions. Partial or complete HIV care interruption was reported at over 80% of HIV clinics in SC, with particularly high rates of interruptions in areas with many uninsured people [3]. Social distancing policies, such as the SC ‘work-or-home’ order (enacted from April 6 to May 4, 2020), were implemented to reduce the spread of COVID-19; however, they may have reduced in-person HIV care accessibility [4]. Fragmented and discontinued HIV care jeopardizes the benefits of antiretroviral treatment, including decreased HIV-related co-morbidities, reduced transmission risk, and improved life expectancy [5–9].

To mitigate care interruptions, nearly 99% of HIV care providers in the United States, who receive funding through the Ryan White HIV/AIDS program to support the care of PWH with low incomes (about 60% of South Carolinians living with HIV [10]), offered telehealth HIV care services [11]. However, participation in and benefits from telehealth visits (i.e., remote HIV care visits using an audio and/or video connection) were not distributed equally. People with unstable housing, racial and ethnic minorities, older individuals, non-native English speakers, and uninsured individuals engaged in fewer telehealth encounters or experienced more challenges [12].

Despite the evidence of unequal telehealth uptake among PWH, the decision-making processes to determine who received telehealth or in-person care are under-researched. General guidance on selecting HIV care visit types remained unspecific throughout the COVID-19 pandemic. The absence of clear, evidence-based criteria shifted the decision burden to HIV care providers. Limited literature suggested that HIV care providers discuss client preferences and their ability to use telehealth [13] or exercise judgement to weigh a client’s medical need for a hands-on exam against their risk of COVID-19 infection [14]. In a previous study, we found that the decision processes varied across organizations; ranging from accommodating client preferences to provider-side decisions based on clinical characteristics [15]. Understanding the underlying and often implicitly applied criteria that were used to make HIV care visit type decisions during the COVID-19 pandemic is crucial to inform evidence-based guidance on the appropriate use of telehealth in HIV care beyond the pandemic. Hence, this study qualitatively assessed which decision criteria and processes determined the HIV care visit type used by PWH and HIV care providers from SC during the COVID-19 pandemic.

## Methods

A qualitative study design following grounded theory approach was used to capture decision-making criteria and processes for telehealth HIV care during the COVID-19 pandemic. The grounded theory qualitative study design was used to develop a conceptual framework of visit type decisions using multiple criteria that was based in the qualitative data. In-depth interviews (IDIs) with PWH and HIV care providers asked open-ended questions to collect rich qualitative data to assess the decision criteria and their consideration in the decision process to determine HIV care visit types. A detailed description of the qualitative methods used in this study has been reported previously in accordance with the Standards for Reporting Qualitative Research guidelines [16,17]. Key information are described below.

### Ethics statement

The study protocol was determined to be exempt from review by the Institutional Review Board of the HIV care providing institution (henceforth “HIV clinic”) participating in this research (#1883275). Interviewees consented verbally to participation in IDIs and the audio recording of interviews.

### Study participants

Interviews were conducted with HIV care providers and PWH receiving care from a large HIV clinic in SC. Adult PWH with an active antiretroviral therapy prescription who received care from the participating HIV clinic were eligible to participate. PWH were selected following a stratified purposeful sampling strategy to ensure a diverse sample in terms of gender, race and ethnicity, and residence [18]. PWH were recruited through a combination of provider referrals, snowball sampling, and study flyers posted in waiting areas of the HIV clinic. Providers were purposively recruited in staff meetings and through snowball referrals and eligible if they (a) were non-trainee healthcare providers, and (b) cared for PWH on a regular basis.

Data saturation guided the sample size. Evidence suggests that data saturation can occur within twelve interviews, with primary themes arising as early as six interviews [19]. We stopped data collection after 28 interviews as we had reached saturation (i.e., additional interviews did not introduce new themes).

### Data collection

Separate interview guides for PWH and providers explored interviewee experiences with telehealth during the COVID-19 pandemic. The guides covered PWH’s and providers’ willingness to use telehealth, their perceived and experienced barriers to telehealth, decision-making processes and criteria for telehealth and in-person HIV care during the pandemic, and perspectives on telehealth in HIV care in the future (Table 1). All interviewees completed a short quantitative supplemental survey to assess basic socio-demographic characteristics.

**Table 1:** Sample questions for interviews with people with HIV and HIV care providers in South Carolina, 2022.

Domain	Sample questions	
	People with HIV	HIV care providers
Telehealth HIV care	Can you please describe the details about your HIV cares services by phone or video including …? … technology used … internet access/plan … place accessing visit … privacy at that place How satisfied or unsatisfied were you with your HIV care services by phone or using video? Please explain why.	Please tell me more about your experiences with telehealth visits. How satisfied or unsatisfied are you with telehealth in HIV care? Please explain why.
Visit type decision-making	How did you and your provider decide if a visit at the clinic or over the phone or video is best for you?	How did you and your patients decide which visit type is best for them?

### Implementation and data processing

IDIs were conducted in-person in a private room at the HIV clinic or virtually via HIPAA-compliant teleconferencing software between July and December 2022. Interviews were audio recorded and transcribed verbatim using Microsoft’s 365 Word online version (Microsoft Corporation, Redmond, WA). To ensure accuracy and privacy, transcripts were reread and corrected if necessary, and names and other identifying information were removed from the transcripts.

### Data analysis

A thematic analysis approach was used to explore and synthesize constructs within the main domains of interest [20,21]. Provider and PWH interviews were coded separately. Transcripts were coded line-by-line in NVivo (QSR International, Burlington, MA) by two members of the research team, using a combination of a deductive and inductive approach. Pre-developed structural coding categories were developed based on the research question and topics covered in the interview guide (deductive ‘codebook thematic analysis’), yet the codes within categories were inductively generated as they arose from the data during the coding process (‘reflexive thematic analysis’) [21]. Codes were then grouped and categorized to identify themes and subthemes related to the decision-making criteria or describing the decision process. Results are accompanied by selected representative, verbatim quotes to illustrate key findings. Quotes presented in this manuscript are exemplary to illustrate the essence of the reported findings and not the frequency that they were mentioned by interviewees. Quotes have been edited to increase clarity. For example, repeated words or non-lexical utterances were removed. Participant characteristics were analyzed using descriptive statistics in Stata (Version 16, StataCorp, College Station, Texas).

## Results

### Sample characteristics

We conducted 28 IDIs with 18 PWH and 10 HIV care providers (see Table 2). Most PWH were female (61.1%), Black or African American (66.7%), and not of Hispanic or Latinx descent (77.8%). PWH had a mean age of 52 years (SD=9.8). The majority lived in an urban area (77.8%) and had public health insurance (61.1%). Most HIV care providers were female (70%), white (50%), not of Hispanic or Latinx descent (90%), with a mean age of 43.3 years (SD=8.9). Most PWH (11 out of 18) used telehealth for HIV care services during the COVID-19 pandemic. Of the remaining PWH, 5 reported that telehealth was never offered or discussed, and 2 reported that they refused to do telehealth. All providers had used telehealth in HIV care ranging from frequent telehealth encounters to fewer than five telehealth encounters.

**Table 2:** Sample characteristics of people with HIV and HIV care providers from South Carolina, 2022.

Characteristic	People with HIV (N=18)	HIV care providers (N=10)
	N (%)	N (%)
Gender		
Female (vs. male)	11 (61.11)	7 (70)
Age		
in years (mean [SD])	52 (9.82)	43.3 (8.92)
Race		
Black or African American	12 (66.67)	3 (30)
White	4 (22.22)	5 (50)
Asian	–	1 (10)
Mixed race	1 (5.56)	–
Prefer not to answer	1 (5.56)	1 (10)
Ethnicity		
Hispanic or Latinx	1 (5.56)	–
Not Hispanic or Latinx	14 (77.78)	9 (90)
Prefer not to answer	3 (16.67)	1 (10)
Place of residence		
Urban (vs. rural)	14 (77.78)	n/a
Education		
Less than high school	2 (11.11)	n/a
Highschool diploma or GED	9 (50)	
Some college	1 (5.56)	
Any college degree or higher	6 (33.33)	
Health insurance^a^		
AIDS Drug Assistance Program	7 (38.89)	n/a
Public insurance	11 (61.11)	
Private insurance	5 (27.78)	
No insurance	1 (5.56)	

Table adjusted from [17];

^a^Multiple responses possible

GED, General Educational Development Test; HIV, human immunodeficiency virus; n/a, not applicable; SD, standard deviation

### Decision criteria for telehealth and in-person HIV care

**Fig 1:**
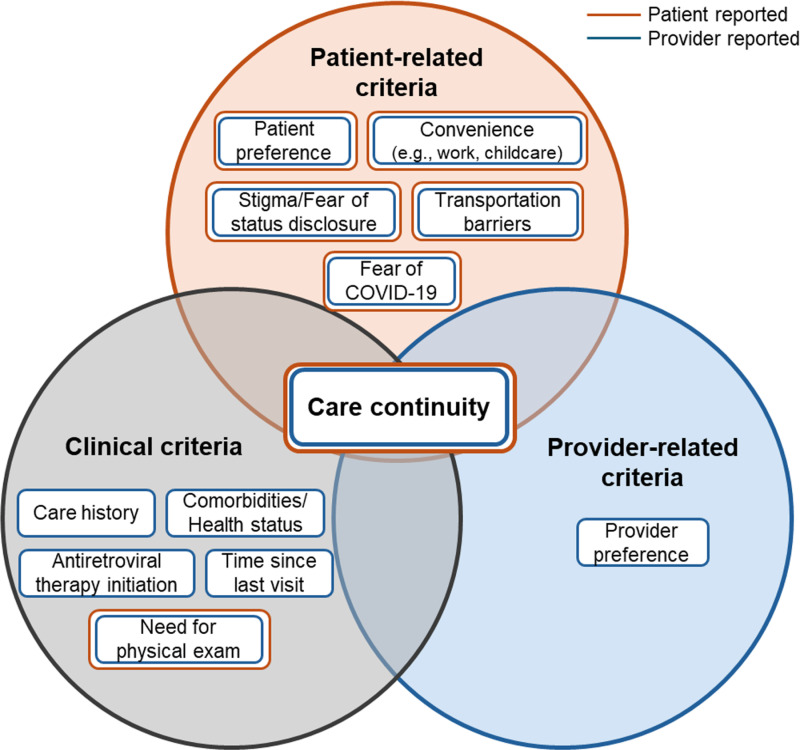
Decision-making criteria for telehealth vs. in-person HIV care.

Telehealth was used for a wide variety of services, including routine HIV care services, medication refills, interim calls to assess PWH’s well-being and care needs, mental healthcare services, case management, primary care, and obstetric and gynecological care. To guide decisions about in-person vs. telehealth HIV care visits, interviewees reported decision-making criteria within four emerging domains: (1) Patient-related criteria, (2) Clinical criteria, (3) Provider-related criteria, and (4) Care continuity (Fig 1).

#### Patient-related criteria.

Patient-related decision criteria included PWH’s preference for either visit type. PWH described that their preference was shaped by their expected level of connection with their provider and their technological savviness. One PWH described,

“it’s a little more personal and, you know, I don’t mind the phone thing. I’m not whole big tech savvy, so therefore it’s just easier for hands on for me with them that I feel like the care is so much better when you’re in person” (PWH interview #108).

Providers and PWH reported that the fear of COVID-19 exposure was impacting visit type decisions. One PWH explained,

“I didn’t really want to come in because of COVID [...] I didn’t like wearing a mask, but I had to. I would wear a mask anywhere I go, and I didn’t trust being around people ‘cause the people that refuse to wear masks” (PWH interview #115).

Interviewees further mentioned that they considered the convenience of telehealth, transportation barriers, stigma and fear of HIV status disclosure in their visit type decision-making. One provider described the transportation and logistical challenges one of their patients faced when coming to an in-person visit, “if she wants to come to an appointment, she’s got to load up her two small children and [get] an [transportation service name] or a taxi […] and that’s just such a pain” (Provider interview #003). Another provider explained how stigma and fear of being seen at an HIV clinic can impact visit type decisions,

“we have some patients that get really nervous, really anxious coming into the clinic because they worry that someone may recognize them and they don’t want their status, you know, disclosed. So, if I notice that with the patient, I do offer, ‘hey, let’s do telehealth visits’. […] It decreases their stress levels coming to the clinic” (Provider interview #007).

Barriers to in-person HIV visits, patient preference, and convenience emerged as patient-related criteria that were factored in visit type decisions to meet individual client needs.

#### Clinical criteria.

Clinical criteria impacting visit type decision-making were primarily reported by providers. Providers explained that they preferred in-person visits for new patients, the initiation of antiretroviral therapy, and when the PWH’s last visit was more than one year ago. One provider described that,

“with our new patients, very new diagnosed patients, their first visit is definitely in the office. […] It’s very important because the assessment is very different. Certain things with a new diagnosis patient, you know less. Say if, if they have oral thrush, if they have a rash on their body, sores on their hands, some new diagnosis patients have those. […] And if they have those symptoms I would do further tests. Whereas with telehealth I may not be able to get a good, you know, thorough assessment” (Provider interview #007).

Other reasons indicating a need for in-person care were a person’s health status including comorbidities, medication side effects, and other medical concerns that may require in-person exams or lab testing. In contrast, providers reported that telehealth may be most suitable for stable and established patients with a history of strong medication adherence and who are otherwise healthy. One provider described that,

“telehealth is perfectly appropriate [… for] patients who don’t have comorbidities, their labs have been consistently good, they’re taking their meds, they’re compliant. I think that’s perfectly appropriate and a great way to use some resources that we have and also just keep people [safe] ‘cause COVID still exists” (Provider interview #002).

Clinical criteria such as the need for a physical exam, a PWH’s health status and care history were considered when deciding on their HIV care visit type.

#### Provider-related criteria.

Providers’ preference was reported to impact the visit type decision-making. Most providers reported that they prefer in-person HIV care services. One provider explained that telehealth “will probably never be my cup of tea. It’s not my favorite mode ‘cause I just prefer a good old traditional in-person [visit]” (Provider interview #003) yet highlighted that they try to accommodate PWH’s needs when appropriate.

#### Care continuity.

While interviewees reported a variety of decision-relevant criteria, the ultimate goal in their decision-making was to ensure that PWH received continuous HIV care services. Interviewees shared their individual trade-offs between personal preferences and care continuity when considering telehealth vs. in-person care. One PWH said, “I would prefer in-person. I mean, over anything, but if I ain’t got no other choice, then it’s better than nothing” (PWH interview #105). Another person explained,

“as long as I was still getting my care for HIV management, as long as I was still getting my meds for HIV, I was good, you know. ‘Cause your HIV, you have to be on top of [it …], I don’t ever want it to go to full blown [AIDS]” (PWH interview #116).

Care continuity was also reported to supersede hesitancy toward telehealth and clinical factors, as one provider explains

“I don’t like telehealth, you know, and that part is kind of inconvenient, but people like it. […] Even if I don’t think it’s like the best for them, […] is it worth saying no, because it’s just a preference maybe, and it’s a resource we can offer” (Provider interview #003).

Another provider explained that they

“would do it [telehealth] if they couldn’t come, like, let’s say they had no car, they had a physical reason why they, say they had a recent fall or something and they couldn’t come. […] I’ll do it as a courtesy. I’ll do that. If I didn’t know how to do it, I’ll do it on the phone. Or I’ll just call them and check on them and not even bill for the visit. Whatever works for them, works for me” (Provider interview #009).

Care continuity was an important consideration in the visit type decision process and emerged as the overarching criterion for visit type decisions. Interviewees described their internal processes of balancing care continuity and other decision criteria and that care continuity was prevailing other criteria.

#### Decision-making processes and involvement for telehealth and in-person HIV care

Most PWH reported that they were included or took the lead in the visit type decision process. One person described that, “they left it up to me, whichever I prefer” (PWH interview #116). Most providers agreed and described having individual conversations to guide the visit selection. One provider explained that “it was personal preference. […] We tried not to mandate anything specifically for the patients“ (Provider interview #001). Others emphasized the importance of shared decision-making as “if you force people to do something that they don’t agree to or don’t want to do, then that’s where you have challenges. […] So, people had options” (Provider interview #010). Providers also highlighted that having visit type options and being involved in the decision process empowers PWH to take a more active role in their HIV care.

However, not everyone felt sufficiently included in the decision process, that either their provider or the clinic decided which visit type they received. One person said, “it was decided for me to do telehealth or not see anybody at all” (PWH interview #105). This sentiment was echoed by a provider who perceived that COVID-19-related policies, including the reduced in-person visit volume at the beginning of the pandemic, forced them to do telehealth. Notably, interviewees who felt low agency in the visit type decision process reported to be unsatisfied with telehealth in HIV care.

Across PWH and providers, most interviewees reported that they were initially hesitant to do telehealth and perceived it as a makeshift rather than an alternative care format. Interviewees explained how they weighed decision criteria to evaluate visit type options. A PWH described their thought process on weighing their personal preference, the feasibility, and benefits of telehealth,

“even though I didn’t like [telehealth], it was doable […] and it could solve the problems or if I had an issue, I could go to over the phone but it was just different. You know, being an older person, I’ve never had to do it that way and it was new. And I don’t take new things lately not too easy. Change is not a good thing for me” (PWH interview #107).

A provider shared how they considered their own preference and their clients’ needs, “It’s not for me unless the patient really cannot come in” (Provider interview #005).

Among PWH who had not used telehealth, reasons to refuse telehealth included a preference for in-person care and suspecting that telehealth would feel impersonal, offer lower quality of care, and impair their ability to express their questions and medical concerns. One person described their reason to refuse telehealth,

“because it’s been my personal feelings that that one-on-one contact with my physician, knowing them very well, them knowing me well and just that personal contact, just feeling more apt to express […] what’s going on with me […] versus, you know, doing it over the video, or, you know via telephone, the telehealth” (PWH interview #118).

Another person said, “just talking on the phone I don’t feel like I can get the care that I need” (PWH interview #117). Other PWH reported that they refused telehealth due to lower digital literacy, living in a rural area and not having a private space at home to do telehealth. One person shared, “I’m not real savvy with that phone. I remember I tried to do a [video call software name; …]. I just can’t do it” (PWH interview #113).

Providers reported that they had to refuse and reschedule telehealth visits due to privacy and safety concerns, after long visit gaps, and for the first visit with new patients. A provider explained,

“I had patients calling, asking for virtual visits, but they haven’t been seen in the clinic for over a year. So, I did decline that because I need to see you in the office, because I want to do a physical assessment” (Provider interview #007).

Most PWH reported that they were included in the visit type decision and there was agreement between providers and PWH on the importance of shared decision-making. The reasons for refusing telehealth differed between PWH and providers. PWH refused using telehealth based on factors related to their care experience, while providers reported privacy concerns and a person’s care history.

#### Virtual care satisfaction

Reported virtual care satisfaction varied across interviewees and changed within interviewees over time. Satisfaction levels ranged from very satisfied to unsatisfied. A PWH shared that they were “most satisfied. Everything is good. […] I ain’t got no complaints” (PWH interview #110). A provider echoed this and shared,

“I am satisfied with telehealth. Telehealth to me, is just another way of providing services and care and it does not stop. I mean, care goes on whether we have telehealth or not. It’s just one more way that we allow people to receive services” (Provider interview #010).

Some PWH and providers explained that their virtual care satisfaction increased over time. A provider said that they “almost like [telehealth] now” (Provider interview #004); a PWH explained that “when I first started, I wasn’t happy at home and then I kind of like got over myself. […] Let’s just do what has to be done to stay alive” (PWH interview #107). Some providers were undecided and explained that their satisfaction with telehealth “depends on the situation and the patient” (Provider interview #001). Few PWH and providers reported that they perceived telehealth as unsatisfactory. One PWH described “I really didn’t like it. I like the fact that I need you to look at me. If I got questions, […] I know I can ask him on the computer, but it just […] didn’t work for me” (PWH interview #102).

While virtual care satisfaction varied across PWH and HIV care providers, most participants who were excluded from the visit type decision process perceived their telehealth visit as unsatisfactory or very unsatisfactory. A provider who reported that the HIV clinic policy left no other option than doing telehealth in the beginning of the pandemic shared, “I’m not […] satisfied you can tell that. […] I feel like whatever we billed for is probably not worth it” (Provider interview #009).

## Discussion

This study described detailed and multi-level decision criteria of telehealth or in-person HIV care and the processes how these criteria were applied to make visit type decisions.

Four domains of decision-making criteria emerged from our data using a grounded theory approach: Patient-related criteria, clinical criteria, provider-related criteria, and care continuity, which expand our previously reported decision framework [15]. Our findings further add to the literature by revealing a hierarchy of decision criteria. While patient-related, clinical, and provider-related criteria were all important and appeared on the same level of importance, care continuity superseded these other criteria. Both PWH and providers revealed their strategies and willingness to trade-off personal visit type preferences and clinical criteria to ensure that HIV care was uninterruptedly continued throughout the pandemic. These findings are highly relevant for HIV care practice and align with the importance of continuous antiretroviral therapy (ART) as outlined in the HIV care guidelines [22]. Our framework may inform future guidance on formal recommendations for visit type decision-making as telehealth is used post pandemic. However, quantitative research taking HIV care outcomes into consideration is needed to determine the limits of such trade-offs. Questions to be asked include (but are not limited to): What are the optimal spacing and alternation patterns of telehealth and in-person care to ensure ART success? How do reports of new symptoms or potential ART side effects impact trade-offs/visit type decisions? Which clinical criteria demand an in-person visit?

In addition to a hierarchy of decision criteria we found that providers reported additional decision criteria to those that were reported by PWH. Most clinical criteria and provider preference were reported by providers but not PWH. While the provider’s focus on and having the ability to consider clinical factors beyond the upcoming visit is not surprising, the divergence of decision factors being reported highlights the importance of shared decision-making to ensure the suitability of telehealth.

In our study sample, there was a consensus that visit type decisions need to be made in collaboration between PWH and their providers within a shared decision-making framework supporting the guidance provided by Smith et al. [13]. However, some interviewees stated that they were not included in the decision-making process. As this study assessed decision processes during the COVID-19 pandemic, we acknowledge that, especially in the beginning of the pandemic, decision involvement was limited due to strict und unprecedented public health measures in response to COVID-19. Notably, PWH and providers who reported that they were not included in the decision process reported low virtual care satisfaction. This finding supports the need for the systematic and intentional inclusion of PWH in their HIV care-related decision-making processes, as shared decision-making has been linked to beneficial HIV care outcomes, including care satisfaction [23].

In contrast to the agreed upon shared decision-making approach, scheduling healthcare visits often occurs between the client and a scheduling coordinator or through electronic health records (EHR) systems. Scheduling systems and their functionality vary between healthcare systems hampering streamlined solutions. Current processes may not include a real-time exchange of information between the client and their care team [24]. To allow for more collaborative visit type decision-making, new strategies need to be implemented in scheduling systems and EHR. For example, EHR systems could include a matching feature where the provider and client can each state their preferred visit type for an upcoming visit. If the preferred visit type does not match, a consensus process could be initiated to find the individually best visit type.

Another factor to consider when scheduling care visits that was not mentioned by our study participants, but needs to be discussed, is health insurance coverage of telehealth. Health insurance coverage and sufficient reimbursement is crucial for clients and HIV care organizations. This study sought to understand the decision criteria and processes that were applied to determine visit type during the COVID-19 pandemic during which policies such as the CARES act and state-level policies ensured telehealth coverage and reimbursement by several funding agencies [25,26]. As many of these policies provided temporary solutions, telehealth coverage and reimbursement rates need to be considered in the future as telehealth policies change and to avoid unexpected cost for clients and loss of revenue for HIV care organizations.

Despite the severe impact of COVID-19 on PWH and their HIV care, our study findings indicate that the implementation of telehealth to provide continuous high-quality care to PWH was successful. While there was initial hesitancy toward telehealth in HIV care, and it was described as a makeshift when no other care options were available, overall, most telehealth users were satisfied with the virtual HIV care they provided or received. These findings align with other studies describing telehealth satisfaction [27,28], and support the need for continued availability of telehealth HIV care options for PWH. Nevertheless, the COVID-19 pandemic was an emergency requiring the rapid implementation of telehealth in HIV care, the continued suitability of telehealth for long-term use and its impact on HIV care satisfaction and outcomes needs to be monitored. Due to limited availability of validated measures to evaluate telehealth HIV care satisfaction and outcomes across disciplines [29], it is important to develop HIV-specific measures to assess telehealth satisfaction and outcomes.

## Limitations

This study is subject to several limitations. First, the generalizability of the study findings may be limited due to the small sample of PWH and HIV care providers from a single HIV clinic. Second, despite the use of sampling quotas to ensure the inclusion of diverse PWH, perspectives of rural and non-Hispanic white PWH may be underrepresented. However, we note that data saturation was reached, indicating that in our study population the study sample was adequate. Third, interviews took place more than two years after the onset of the COVID-19 pandemic which may have induced some recall bias of telehealth experiences in the beginning of the pandemic.

## Conclusions

Telehealth in HIV care was an essential and useful tool for mitigating in-person HIV care interruptions due to the pandemic. Decision-making processes to determine HIV care visit types considered criteria across multiple domains (patient-related, provider-related, clinical decision criteria, and HIV care continuity). HIV care continuity superseded other decision criteria. To guide future telehealth use, shared decision-making is needed to weigh patient-related, provider-related, and clinical decision criteria and ensure care continuity.
